# Smart integration of cold plasma stream and surface discharge with ns laser ablation for composite nanomaterial

**DOI:** 10.1186/s11671-024-04034-4

**Published:** 2024-05-27

**Authors:** Hafiz Muhammad Akhtar, Muhammad Latif, Mahtab Ahmad khan, M. Abdullah, Taj Muhammad Khan

**Affiliations:** 1 National Institute of Lasers and Optronics College, Pakistan Institute of Engineering and Applied Sciences, Nilore, Islamabad 45650 Pakistan; 2 Department of Physics, Federal Urdu University of Arts, Science and Technology, Islamabad, 44000 Pakistan; 3 Tokamak Plasma Research Institute, Nilore, Islamabad 45650 Pakistan

**Keywords:** Composite nanomaterial, Surface and interfaces, Atmospheric pressure plasma, Surface discharge, FESEM, Raman scattering

## Abstract

In this paper, smart integration of cold dielectric barrier discharge (DBD) plasma in various geometrical arrangements with laser ablation at atmospheric pressure for nanomaterial was described. A composite Co:ZnO target was ablated in an airflow by a nanosecond (ns) laser (wavelength: 1064 nm, pulse duration: 30 ns) using fluence of 5 J-cm^−2^ at a repetition rate of 10 Hz. The nanomaterial produced under vertical and oblique plasma streams, surface discharge and gas flow, were compared. Utilization surface discharge markedly improved the material adhesion by altering surface intrinsic behavior, inducing anticipated surface energy activation, chemical changes, and the formation of a densely packed solid structure. Under all conditions, the material consistently retained its crystalline nature, elemental composition, and ultraviolet emission characteristics. These preliminary findings hold promise for additional research, suggesting avenues for making complex materials in a flexible environment. Such new advancements could facilitate applications in the biomedical, catalysis, pharmaceutical, and surgical device domains.

## Introduction

Material processing and fabrication at ambient conditions by harnessing short and ultrashort laser light is a highly impactful area of research. Nowadays, it finds widespread applications in both large-scale manufacturing of everyday products and very specialized uses. It involves the interaction of a laser pulse with fluence above the ablation threshold with the target material which leads to the formation of a high density plasma plume, spatially restricted to a couple of millimeters by the ambient air above the irradiated target surface [1, 2]. This controlled plasma plume typically moves at a relatively slow pace, around 500 ms^−1^ at the laser irradiance of 1.4 J-cm^−2^. This controlled movement is essential for the efficient collisional condensation of the ablated material with the ambient gas, enabling the rapid creation of nanomaterials within a few microseconds (µs) [1, 3]. It is worth mentioning that during both nanosecond (ns) and femtosecond (fs) laser ablation processes, the collisional condensation of the ablated material can lead to the formation of nanoparticle (NP) and/or microparticle aerosols, depending on the specific ablation conditions. Consequently, the contribution to the formation of microscopic clusters in the deposited structures could arise from one or both types of particulates. A previous report has described such an aspect by processing the ablated material with the cold plasma jets of argon and helium [4]. The gas condensation method involving laser ablation in a contained environment appears to be an effective fabrication method for nanostructured films for diverse applications in the active fields of surface-enhanced Raman spectroscopy (SERS), catalysis, and solar cells [1, 2, 4, 5]. Recently, T.M. Khan et al. [1, 4, 6] explored an innovative approach by integrating a nonthermal plasma jet with laser ablation at atmospheric pressure, wherein the plasma jet was allowed to directly interact with the ablation plume induced by a ns laser, allowing the transfer of particle aerosol for deposition on a distant substrate. The morphology, spatial distribution, and surface coverage were greatly controlled by the interplay of the jet length, substrate position, number of laser shots and fluence value. A gas flow assists mechanism was used to entrain the NP aerosols for deposition [1, 2, 4]. In these studies, localized and redeposition of the particles on the surface of the irradiated target was greatly minimized and the surface coverage and the particle size were made tunable for chemical detection. The use of a nonthermal plasma activated surface, achieved through surface dielectric barrier discharge (SDBD), is of particular interest due to its ability to favorably interact with the deposit lands on the surface and preclusion of the formation of oxidation states on the deposited surface. SDBD plasma is essentially a cold plasma, generated through the ionization of gas at or near ambient temperatures. The discharge that ionizes a thin plasma sheet near a dielectric surface at high frequency and high ac voltage (typically kV/kHz), making it a valuable tool for material’s deposition [7]. The deposition of a nanomaterial on a surface with discharge applied and or plasma stream in conjunction with the ablation process could be of particular interest for transparent oxides with tuneable properties for photo-catalysis, solar cell and other optoelectronic devices.

Composite conducting transparent oxides (TCOs) of Co:ZnO has sparked intense research efforts for its wide range of promising applications, such as piezoelectric sensors [8], optoelectronic devices [9], photovoltaics [10], biosensors [11], and transparent, and spin electronics devices [12, 13]. A detailed study of defects in Co-doped ZnO nanostructures, prepared via a chemical route, was previously conducted [14]. However, working with pulsed laser ablation at atmospheric pressure, to create complex structures of transparent oxides and their composites with desired features still presents significant challenges. Nikov et al. [10], for the first time, reported ZnO nanostructures produced by pulsed laser deposition (PLD) in ambient air. This research revealed a profound dependence on wavelength of the laser light across various aspects of the deposited nanostructures such as morphology, composition, and optical characteristics. Specifically, their findings showed that ultraviolet laser ablation led to the formation of NP aggregates and individual NPs. When employing visible light for the ablation process, a highly porous structure composed of aggregated NPs emerges. Conversely, conducting the ablation process with infrared radiation yielded the growth of an intricate 3D structure, primarily composed of sizable nanoparticle aggregates. Utilizing laser ablation of titanium in an air environment, researchers have successfully generated nanoporous titanium oxide (TiO_2_) electrodes [15]. In their work, the authors observed the coexistence of mixed phases, encompassing non-stoichiometric anatase and rutile, with only a minimal presence of titanium nitride (TiN). Nevertheless, the creation of intricate systems with tailored properties remains a formidable task when employing this method. Consequently, the development of composite Co:ZnO nanostructures through atmospheric Pulsed Laser Deposition (APLD) on cold plasma-assisted substrates emerges as a novel and intriguing research direction, building upon prior investigation.

This paper reports on the ns ablation of a composite Co:ZnO target formed by solid state sintering method and subsequently the transport of ablation material and deposition of nanostructures on various substrates in ambient air under two plasma schemes. The deposition process was investigated under two distinct plasma aided settings: (a) with the plasma streams oriented vertically and obliquely, and (b) with gas flow on the surface discharge supporting substrate. It opens up the potential to modify material properties by direct interaction of the plasma stream with the ablation material and/or through deposition onto a substrate with discharge applied: both add new aspects to the underlying study. This approach offers numerous possibilities for expansion to other semiconductor oxides and metals, with potential applications in fields such as solar cells, catalysis, and detection.

## Experimental

**Fig. 1:**
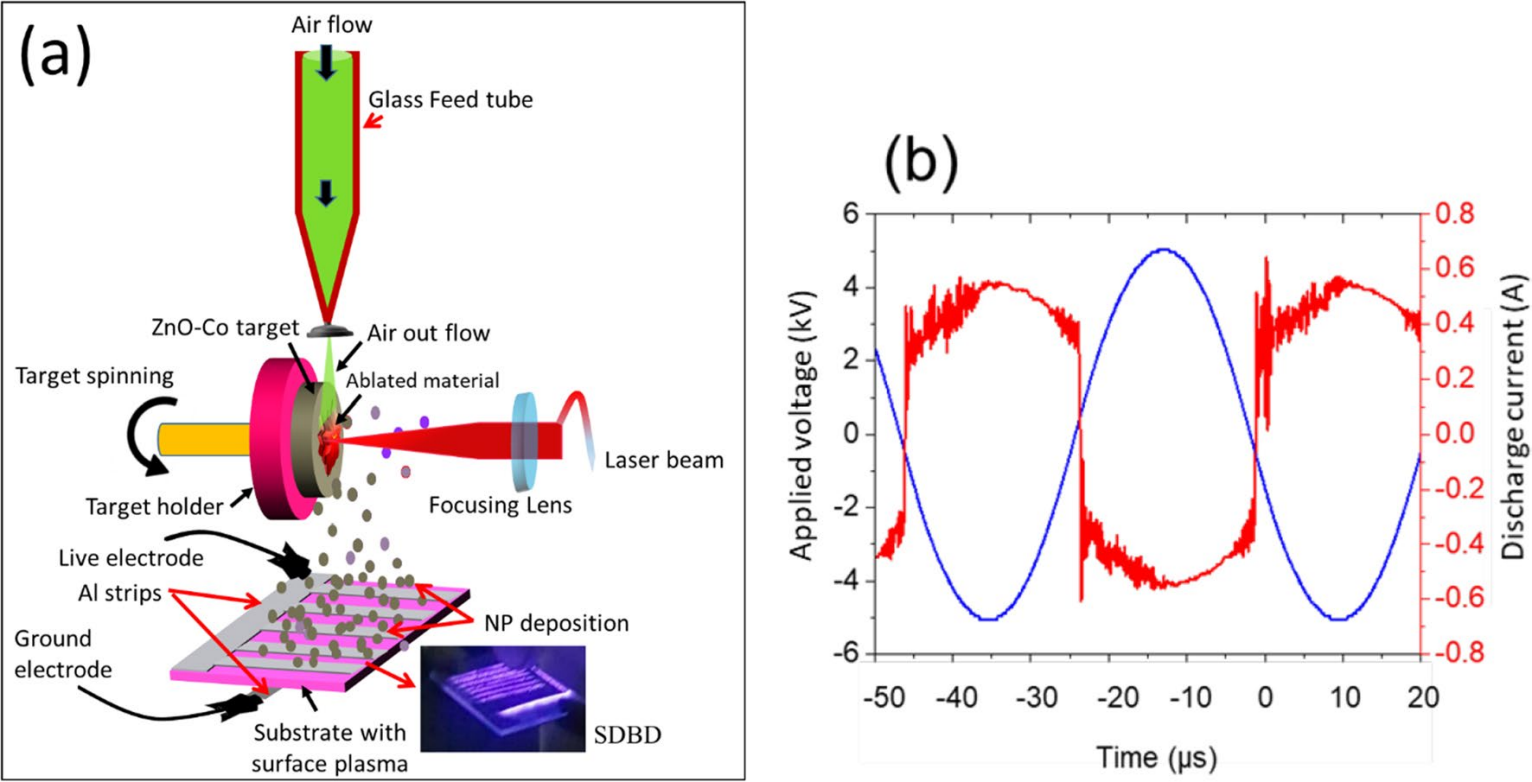
**a** Schematics of APLD used in conjunction with SDBD for the deposition of Co:ZnO nanostructures, **b** current–voltage waveforms of SDBD excited in air on glass surface. The inset photograph indicates the surface discharge excited on glass in ambient air

A composite pellet of Co:ZnO target underwent ablation via ns laser radiation (1064 nm, 30 ns) at a fluence of 5 J-cm^−2^ and a repetition rate of 10 Hz in an airflow (Fig. 1). A plasma stream and surface discharge were produced by a DBD source using a high amplitude ac 3–5 kV at 30 kHz (detailed information about DBDs can be found in our previous reports [1, 4, 6]). The plasma stream was excited within the quartz tube in a single electrode arrangement where a copper wire is centrally positioned and driven electrically by ac voltages. The surface discharge was achieved by wrapping multiple aluminum strips (length 30 mm, thickness 1 mm) on the top and a single strip on the bottom of a glass slide, making plasma active channels on top of the glass with a gap of about 1 mm. The deposition of Co:ZnO nanomaterial with plasma streams both in the vertical/oblique geometries and under the influence of surface discharge within the gap was examined and compared. The characterization of the resulting materials involved the use of field emission scanning electron microscopy (FE-SEM) (Tescan), energy dispersive X-ray spectroscopy (EDS), Raman and photoluminescence (PL) (DongWoo Optron), and X-ray diffraction (XRD) (Equinox 3000). To monitor and measure current–voltage signals, an HV-probe, a resistor (R = 50 Ω), and a digital oscilloscope (Tektronix) were used.

## Results and discussion

Figure 1b illustrates a representative characteristic current–voltage waveforms of the surface discharge, excited by a DBD plasma source within the channels on a glass slide. Compared to the plasma stream (not shown here), the surface discharge generates at a relatively lower amplitude of the applied ac voltage and the discharge activity occurs in both half cycles over a long period of the applied voltage with transient behavior changing remarkably over each half cycle. The discharge in the channels presents visible dark-bluish plasma of multiple spikes, characteristic of a spatially spaced plasma of several discharges and is therefore filamentary in nature. Both positive and negative discharge currents have a series of narrow pulses, and are superimposed, indicating that the discharge exists simultaneously with the diffuse mode on the sinusoidal waveform, which is sometimes called the glow-like filamentary discharge. The positions of the current pulses appearing on the ac voltage indicate ignition voltage of the discharges happen in each half cycle at a particular value of the ac voltage and the magnitude of the current that flows through the gas upon ignition. The current magnitude in SDBD is relatively higher than in the plasma stream, indicating a higher plasma density in the surface discharge.

**Fig. 2:**
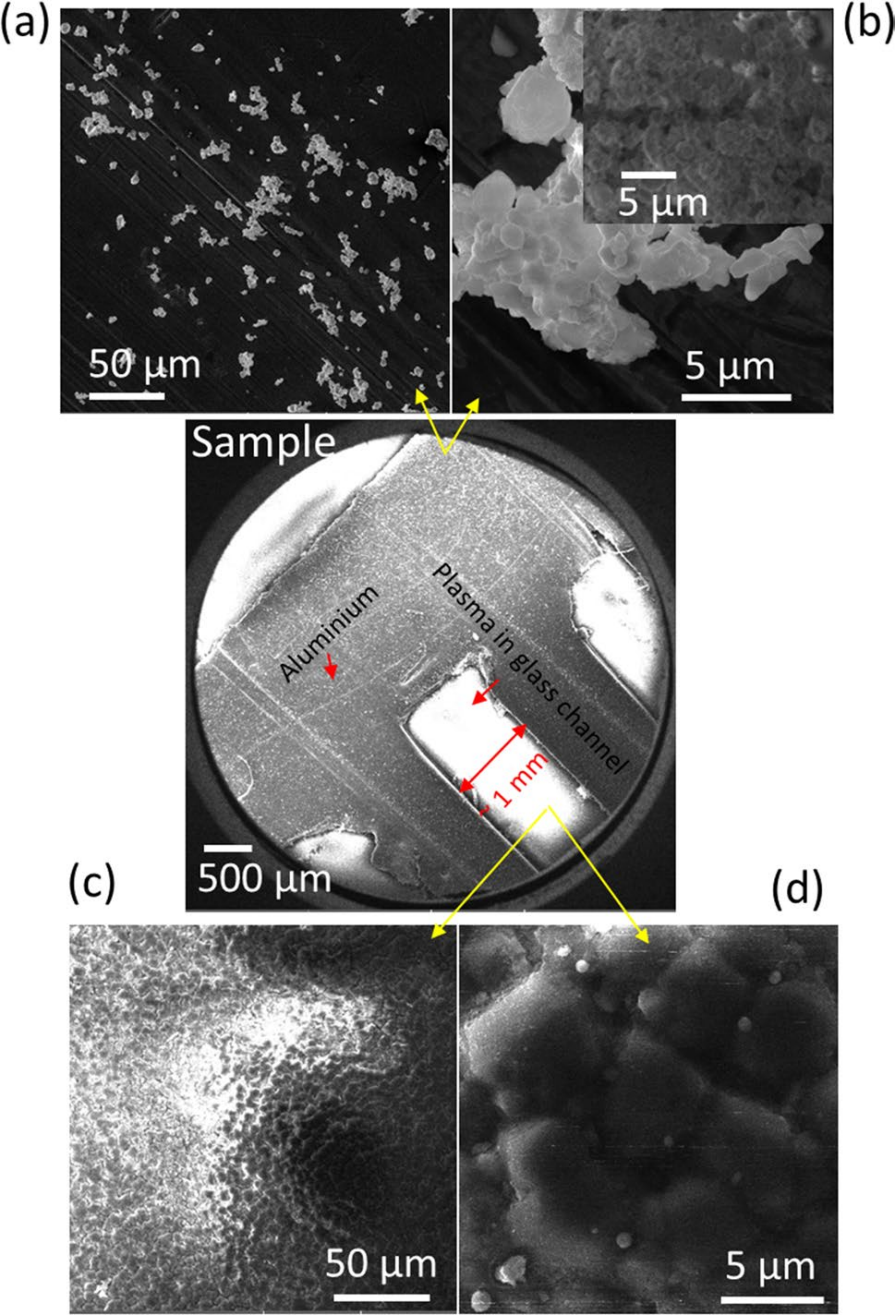
**a**, **b** SEM images of composite Co:ZnO nanostructures on Al strip type electrode, **c**, **d** on glass with the discharge on the surface (SDBD). The substrate distance was 10 mm, deposition time 300 s, and the gas flow was 4 lit per min respectively. Inset in **b** is a representative image of a different sample obtained with a deposition time of 600 s and the rest of the deposition conditions persist

**Fig. 3:**
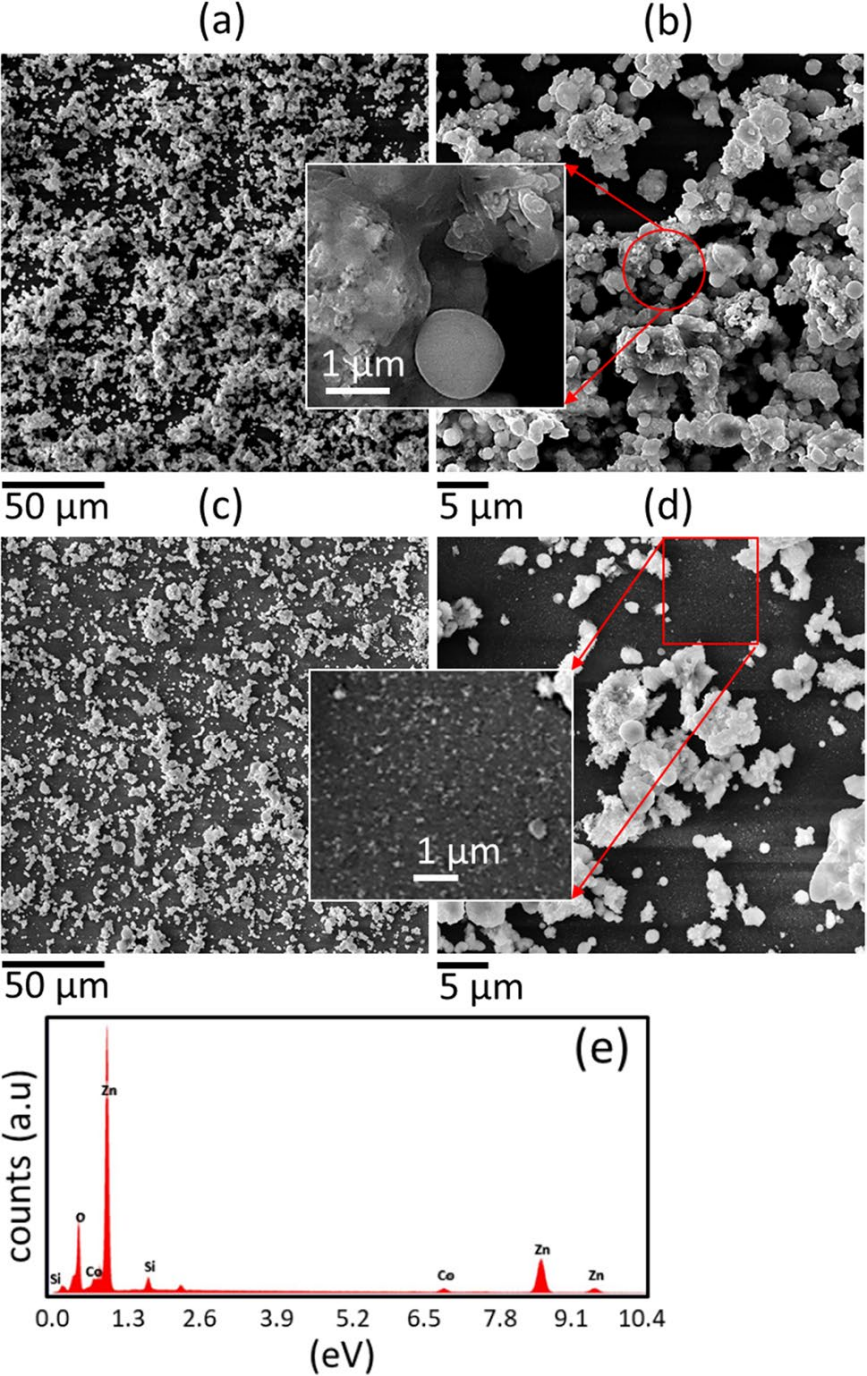
SEM micrographs of composite Co:ZnO nanostructures taken on the densely columnar deposition on Si at two different magnifications obtained with plasma stream in oblique geometry **a**, **b** and vertical arrangement **c**, **d** using ns laser ablation and substrate at 10 mm, deposition time 600 s, and air flow of 4 lit. min^−1^. **e** is the EDX for sample **a** and insets are magnified images of the indicated regions

**Fig. 4.**
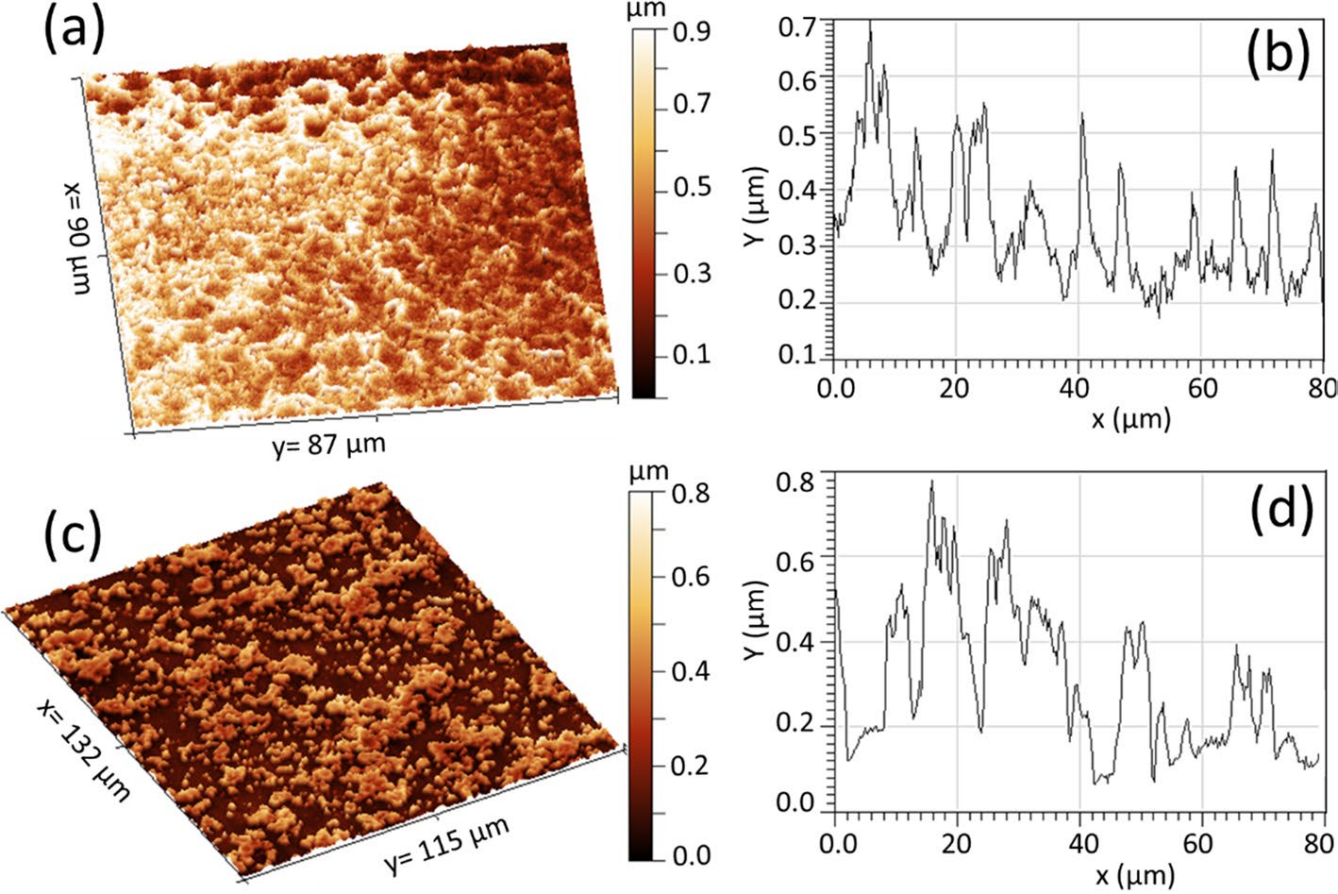
3D images **a**, **c** and surface line profiles **b**, **d** extracted for the nanostructures previously displayed in Figs. 2c and 3c respectively using Gwyddion software

**Fig. 5:**
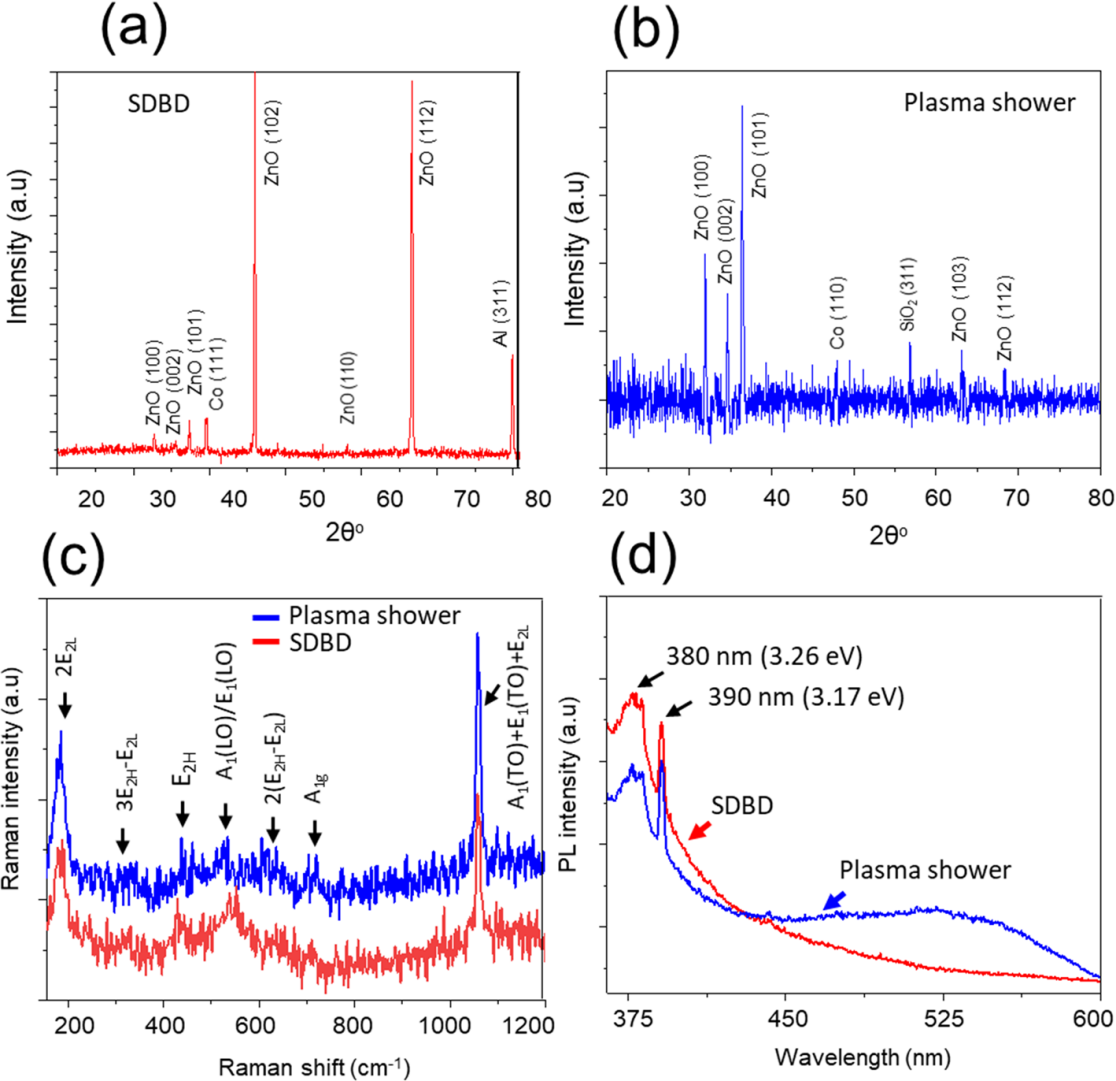
XRD graphs of Co:ZnO nanomaterial obtained with air flow on SDBD activated surface (**a**), deposited with plasma stream in oblique geometry (**b**), and room temperature Raman and photoluminescence spectra (**c**, **d**). The excitation wavelengths used were 532 nm and 325 nm for (**c**) and (**d**) respectively

Figure 2 displays the FE-SEM images of Co:ZnO nanostructures formed on the aluminum strip, acting as an active electrode with no surface discharge on it (Fig. 2a, b) and SDBD driven glass surface (so- called plasma active channels) (Fig. 2c, d). An overall comprehensive SEM image in Fig. 2 illustrates the glass substrate with small active plasma channels and aluminum strips serving as active electrodes to induce surface discharge within the channels. On the glass active plasma channel, the deposited material presents a compact morphology, accompanied by sparsely dispersed spherical structures. This compact formation is attributed to the filamentary structure of SDBD, which generates high-temperature regions, thereby facilitating the merging and diffusion of particle aerosols. These aerosols resulted from the collisional condensation of the ablated material, landing on the plasma sheet's surface. Also, plasma can provide energy to promote nucleation and growth of particles on the surface. This energy facilitates the formation of a closely packed, continuous film as the particles aggregate and adhere to each other. In contrast, the material deposited on the aluminum strip shows widely dispersed clustered structures. This difference suggests that the presence of plasma on the glass surface promotes the formation of a relatively denser structure. The amount of deposit varies with factors such as air flow velocity, the number of laser shots, and the substrate's position. There tends to be a larger deposit in the surface region near the ablated target, especially for extended deposition times (600 s), as indicated in the inset of Fig. 2b. Elemental composition analysis revealed the presence of Zn, O, and Co within the deposit, not presented here. The detection of these elements confirms that the composite Co element which was originally present in the composite target material also retained during the laser ablation and deposition process in the deposited structures. Figure 3a–d show Co:ZnO clusters formed on Si with plasma streams in the oblique and vertical settings respectively. The arrangement used was similar to that shown in Fig. 1a. However, a plasma stream was excited in the gas flow to transfer the ablated material to the substrate with no discharge on its surface. The amount of these clusters was relatively less in the former case because, in the oblique arrangement, the plasma-air stream is partially abstracted by the irradiated target, leading to redeposition on its surface and limited material transfer to the substrate. A distinct region of dense, columnar deposition was noted when the plasma stream was directed obliquely. Though this region exhibited a relatively higher deposition compared to the vertical plasma stream (Fig. 3a, b), the overall deposit was smaller when employing the oblique plasma stream due to the redeposition of ablated material on the target surface. In both cases, the morphology of the nanocomposites consisted of spherical and flower-like nanostructures. The formation of clusters was predominantly influenced by moisture introduced through the airflow, leading to agglomeration. The insets in Fig. 3 depict magnified SEM images of the designated regions. Notably, the vertical plasma stream also led to the formation of small-sized particles within the clustered material, as indicated in the insets of Fig. 3c, d. The composition of the target constituents in the ablated material was confirmed by EDX (Fig. 3e). For the deposited hybrid nanostructures obtained with SDBD and plasma stream in the vertical configuration, the 3-dimensional images and respective surface profile graphs were extracted using Gwyddion software and are described in Fig. 4a–d. These 3D images provide valuable information about the physical morphological alterations and visual adaptations occurring on the deposited surface. These images quantitatively express the surface heights using line profiles. Fluctuations in the surface heights, numerically expressed through the line profiles, signify irregular surface behavior attributed to variations in the size of individual particles and clusters. Primarily, Gwyddion software is utilized to acquire convoluted duplicate images with reduced noise and enhanced contrast, originally captured with SPM and AFM. Additionally, it serves to analyze SEM images, improving manipulation and surface presentation. Through line scans, it facilitates the visualization of height variation across the sample structure, providing quantitative support for the formation of large islands in the coated material. The results of XRD, Raman and PL of the nanostructures are presented in Fig. 5a–d. The XRD spectrograph revealed crystallographic diffraction planes of ZnO, Co constituents, and diffraction peaks originating from aluminum and glass substrates. The Raman active optical phonon modes (Fig. 5c) support the formation of composite ZnO nanostructures [16, 17]. The nonpolar E_2_ modes have two frequencies: E_2_ (high) associated with vibration of the oxygen atom and E_2_ (low) associated with vibration of Zn atoms. The E_2_ modes exhibited changes due to the incorporation of Co into the ZnO lattice. The peak at 670 cm^−1^ (A_1g_) originates from Co-oxide nanocrystals in the composite Co:ZnO. The photoluminescence spectrum (Fig. 5d) displayed two closely spaced emissions spanning the region from 360 to 400 nm. The presence of two closely spaced emissions in the UV region in composite Co:ZnO materials is typically due to the interaction of cobalt ions (Co^2+^) with the ZnO host lattice, resulting in different energy levels and electronic transitions. The first relatively broad and weak intensity near band edge emission, occurring at 380 nm (3.26 eV), is ascribed to Co–O nanocrystals and attributed to the radiative annihilation of excitons [13], while the second sharp and intense luminescence at 390 nm (3.17 eV), is related with crystalline ZnO particles [10, 16]. The ZnO band edge emission at 390 nm originates from the recombination of the free excitons through an exciton-exciton collision process [10, 18]. Additionally, in the sample formed by the plasma stream, a weak emission in the range of 450–600 nm, centered in the green region, was observed, often associated with deep-level defects of oxygen in ZnO nanomaterials [18]. Importantly, this research indicated that the SDBD plasma sample did not exhibit Raman phonon modes and green emissions corresponding to oxygen defects, suggesting effective absorption of oxygen from the surrounding plasma environment. This observation indicates that the plasma process is likely to consume or deplete oxygen-related defects or impurities in the sample. It is understood that cold atmospheric pressure plasma discharges offer an abundant source of reactive oxygen and nitrogen species (RONS) at room temperature, enabling unique surface chemistry. However, further investigation is needed to understand the presence of reactive oxygen species (ROS) originating from the plasma source, its absorption and chemistry on the surface, and to consider reactive nitrogen species (RNS) for a comprehensive understanding of oxygen defect-free ZnO formation with surface-DBD supported PLD.

## Conclusion

In summary, Co:ZnO composite nanostructures were effectively achieved through atmospheric-PLD with cold DBD plasma, employing settings that involved both the plasma stream and surface discharge to aid in the deposition process. A comparison between the vertical and oblique plasma streams revealed that the vertical stream resulted in a more substantial deposition across the substrate, yielding a mixed surface morphology characterized by clusters and smaller particles evenly distributed within clustered regions. Conversely, the oblique plasma stream led to the formation of a localized region consisting of a dense columnar deposit. A significant difference in deposit morphology was observed with SDBD, where the surface discharge facilitated particle inter-diffusion and assimilation, ultimately culminating in the formation of a densely packed solid structure. Comprehensive assessments confirmed the polycrystalline nature and emission properties of the deposit. These results highlight the effective utilization of the unconventional APLD method alongside cold plasma across various geometrical operations to fabricate composite Co:ZnO. This also demonstrates its capacity to create other composite oxides and advanced materials, thereby indicating its versatility for applications in fuel and solar cells, sensor, and catalysis.

## Data Availability

Data underlying the results presented in this paper can be obtained from the corresponding author upon reasonable request.
